# FRAILTY INDEX AT THE BEGINNING OF ELDERHOOD AND HEALTHCARE COSTS AND UTILIZATION OVER 10 YEARS

**DOI:** 10.1093/geroni/igac059.2731

**Published:** 2022-12-20

**Authors:** Jieun Jang, Dae Kim

**Affiliations:** Hebrew SeniorLife, Harvard Medical School, Boston, Massachusetts, United States; Hebrew SeniorLife, Harvard Medical School, Boston, Massachusetts, United States

## Abstract

**Background:**

We assessed whether the frailty index measured at the beginning of elderhood can predict healthcare costs over 10 years in a nationwide Korean population.

**Methods:**

This retrospective cohort study included 215,887 individuals who underwent a standardized comprehensive geriatric assessment at the age of 66 years as part of the National Screening Program for Transitional Ages in 2007–2009 and participants were followed up until December 31, 2019, from the Korean National Health Insurance database. Frailty status was defined based on a 39-item frailty index: robust (< 0.15), pre-frail (0.15 to < 0.25), frail (≥0.25). Generalized linear model was used to examine any changes in healthcare cost among pre-frail group, frail group following 10 years from the age of 66 years, relative to changes in healthcare cost of the robust group. This study constructed an interaction term between the frail group and age.

**Results:**

Frailty status at age 66 years was associated with an increased annual total healthcare cost per NHI beneficiary (robust * age vs frail * age: β = 89.5, SE = 4.0, P < .0001), annual inpatient healthcare cost per NHI beneficiary (robust * age vs frail * age: β = 70.3, SE = 4.1, P < .0001) over 10 years, but not significant in annual outpatient cost per NHI beneficiary after adjusting for frailty category, demographic factor, socioeconomic factor, and time fixed effect. Conclusions: The frailty index at the age of 66 years was associated with an accelerated increase in healthcare costs over 10 years.

